# Peripheral Inflammation Acutely Impairs Human Spatial Memory via Actions on Medial Temporal Lobe Glucose Metabolism

**DOI:** 10.1016/j.biopsych.2014.01.005

**Published:** 2014-10-01

**Authors:** Neil A. Harrison, Christian F. Doeller, Valerie Voon, Neil Burgess, Hugo D. Critchley

**Affiliations:** a Department of Psychiatry, Brighton and Sussex Medical School, Falmer, United Kingdom; b Sackler Centre for Consciousness Science, University of Sussex, Falmer, United Kingdom; c Donders Institute for Brain, Cognition and Behaviour, Radboud University Nijmegen, The Netherlands; d Department of Psychiatry, University of Cambridge, Cambridge, United Kingdom; e Institutes of Cognitive Neuroscience and Neurology, University College London, London, United Kingdom

**Keywords:** Alzheimer’s disease, imaging, inflammation, memory, parahippocampus, PET

## Abstract

**Background:**

Inflammation impairs cognitive performance and is implicated in the progression of neurodegenerative disorders. Rodent studies demonstrated key roles for inflammatory mediators in many processes critical to memory, including long-term potentiation, synaptic plasticity, and neurogenesis. They also demonstrated functional impairment of medial temporal lobe (MTL) structures by systemic inflammation. However, human data to support this position are limited.

**Methods:**

Sequential fluorodeoxyglucose positron emission tomography together with experimentally induced inflammation was used to investigate effects of a systemic inflammatory challenge on human MTL function. Fluorodeoxyglucose positron emission tomography scanning was performed in 20 healthy participants before and after typhoid vaccination and saline control injection. After each scanning session, participants performed a virtual reality spatial memory task analogous to the Morris water maze and a mirror-tracing procedural memory control task.

**Results:**

Fluorodeoxyglucose positron emission tomography data demonstrated an acute reduction in human MTL glucose metabolism after inflammation. The inflammatory challenge also selectively compromised human spatial, but not procedural, memory; this effect that was independent of actions on motivation or psychomotor response. Effects of inflammation on parahippocampal and rhinal glucose metabolism directly mediated actions of inflammation on spatial memory.

**Conclusions:**

These data demonstrate acute sensitivity of human MTL to mild peripheral inflammation, giving rise to associated functional impairment in the form of reduced spatial memory performance. Our findings suggest a mechanism for the observed epidemiologic link between inflammation and risk of age-related cognitive decline and progression of neurodegenerative disorders including Alzheimer’s disease.

Although previously considered an immune-privileged site, it is now clear that the immune system plays an integral role in many fundamental neuronal processes, including long-term potentiation (LTP) 1, 2, synaptic plasticity (3), and neurogenesis (4), that are critical to learning and memory. In health, immune mechanisms regulate each of these processes and assist in the remodeling of neural circuits that promote learning and memory (5). However, during systemic infection or injury (6), this positive regulatory function is disrupted, resulting in acute memory impairments: When inflammation is severe, cognitive impairment may become persistent (7), and when chronic inflammation is present, age-related cognitive impairment is accelerated (8). Inflammation may drive the rapid progression of neurodegenerative diseases such as Alzheimer’s disease (9).

Structures in the medial temporal lobe (MTL) appear to be particularly sensitive to effects of inflammation. This increased sensitivity may be related to their relatively high receptor and messenger RNA expression for proinflammatory cytokines 10, 11 and their neural connectivity to regions such as the insula (12) that support cortical representations of peripheral inflammatory states (13). Rodent studies emphasized the role of the hippocampus; direct administration of inflammatory cytokines, particularly interleukin (IL)-1, into the hippocampus selectively impaired spatial and contextual memory processes, including radial arm and Morris water maze performance, and contextual, but not auditory-cued, fear conditioning 5, 14, 15. Similarly, over-expression of IL-1 messenger RNA within the hippocampus is associated with delayed acquisition of spatial memory on the Morris water maze task (14). For synaptic plasticity underlying the encoding and recall of memories, LTP is arguably the key neuronal mechanism. The cytokine IL-1 compromises both hippocampal and dentate gyrus LTP 1, 17, 18 and may mediate both age-dependent decreases in LTP (19) and the modulation of LTP by Aβ amyloid (20). Cytokine-induced inhibition of neurogenesis within the dentate gyrus is also alleviated by the microglial inhibitor minocycline (4). Together, these data highlight the central action of inflammatory mediators (cytokines such as IL-1) on MTL-dependent memory processes.

Inflammatory challenges administered outside the central nervous system also induce IL-1 expression within brain regions, including the MTL (21). Peripherally induced inflammation also replicates many of the direct actions of inflammatory cytokines on MTL-dependent memory 5, 22, 23. There are numerous mechanisms through which peripheral inflammation can engender changes in cytokine levels within sensitive brain regions. Circulating cytokines may be actively transported across the blood-brain barrier (24) or activate microglia via the circumventricular organs (25) and vascular endothelium (26). However, local synthesis of IL-1 is suggested by the rapid upregulation of IL-1α and IL-1β gene expression and the central predominance of the short half-life IL-1 isoform in the context of mild systemic inflammatory challenge (21). Vagus nerve afferents show sensitivity to peripheral cytokines (27) and mild inflammatory challenge (28) indicating an additional neurally mediated immune-brain pathway. Central vagus nerve targets show enhanced activity within 2–3 hours of peripheral inflammatory challenge in both rodents and humans 29, 30. Electrical stimulation of vagus nerve afferents results in a rapid increase in IL-1β expression within the hippocampus (31). Humoral and neurally mediated routes may communicate peripheral inflammatory responses centrally to regions supporting memory processes.

These data from animal studies suggest mechanisms to account for human epidemiologic data linking increased peripheral inflammation to accelerated cognitive aging and neurodegeneration. However, it is unknown whether systemic inflammation modulates MTL function in humans. We used an experimental inflammatory model, typhoid vaccination, together with sequential fluorodeoxyglucose (FDG) positron emission tomography (PET) scanning to quantify hypothesized effects of peripheral inflammation on human MTL function and spatial memory. In 20 healthy participants, three FDG-PET scans were performed immediately before and 4 hours and 8 hours after typhoid vaccination or control (saline) injection (Figure 1). After each of the first two scanning sessions, participants performed a spatial memory task in which they learned and then recalled the identity and location of two sets of 16 objects positioned within a virtual reality environment. This virtual reality task is analogous to the Morris water maze (32), which is sensitive to inflammatory effects on object-location accuracy in rodents, and to the hidden tracer task, which is sensitive to lesions in discrete MTL structures in humans (33). Recall of the spatial location and identity of both sets of objects was tested again after the third scan to investigate differential effects of inflammation on early encoding and later consolidation processes. Participants also performed a mirror-tracing procedural memory task to test general effects of inflammation on psychomotor responses and motor learning.

**Figure 1:**
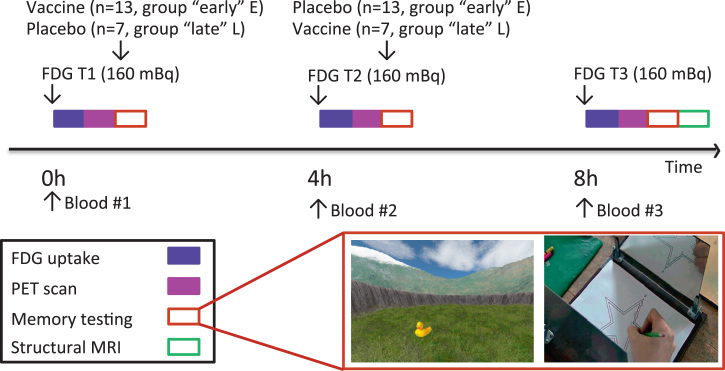
Study timeline. All participants completed three fluorodeoxyglucose (FDG) positron emission tomography (PET) scans. Each scan was preceded by a blood draw and followed by a memory testing session. The “early” inflammation group received the typhoid vaccination after the first PET scan (and saline injection after the second PET scan), and the “late” inflammation group received the typhoid vaccination after the second PET scan (saline injection after the first scan). In the first two sessions (T1 and T2), participants encoded and then recalled the identity and spatial location of two sets of objects (object set 1 and set 2). In the third session (T3), participants recalled the identity and spatial location of the two sets of objects encoded at T1 and T2. The mirror-tracing task was performed at each of the three testing sessions. MRI, magnetic resonance imaging. (Photo credit, copyright WGBH Educational Foundation.)

## Methods and Materials

### Participants

We recruited 20 healthy male nonsmokers (mean age, 24.7 ± 6.8 years old) and screened them for relevant physical or psychiatric illness; all were medication-free. Volunteers who had received typhoid vaccine within 3 years or other vaccine within 6 months were excluded. Participants were advised to avoid caffeinated beverages, alcohol, high-fat meals, and excessive exercise for 24 hours and steroidal or nonsteroidal drugs for 2 weeks before testing. All participants fasted for 8 hours and consumed only water until study completion. Written informed consent was obtained from all participants, and procedures were approved by the Brighton East National Research Ethics Committee.

### Study Design

A randomized, double-blind, repeated measures crossover design was used in which all participants underwent three FDG-PET imaging sessions each separated by 4 hours. After each of the first two scanning sessions, participants randomly received intramuscular injections of either 0.025 mg *Salmonella typhi* vaccine (Typhim Vi; Aventis Pasteur MSD Ltd., Lyon, France) or 0.5 mL normal saline. Of participants, 13 were randomly assigned to the early inflammation group and received vaccination after the first PET scan 1, and 7 were randomly assigned to the late inflammation group and received vaccination after the second PET scan. This study design enabled us to control for nonspecific time effects as well as have sufficient participants (*n* = 13) scanned 8 hours after typhoid vaccination to test late effects of inflammation. After each scan, participants performed a laptop-based spatial memory task and a mirror-tracing procedural memory task that took 35 min to complete. Vaccination or saline injection was given after the PET scan immediately before memory testing; this was done to minimize an already long testing day. We are aware of no data to suggest that peripherally induced inflammation can impair memory at such a short latency, and if this were the case, it would increase the risk of false-negative rather than false-positive findings. A high-resolution inversion recovery echo planar image was obtained to aid image registration.

### Inflammatory Model

We used a *S. typhi* vaccination model known to induce low-grade inflammation without body temperature change (34). Blood (10 mL) was drawn into ethylenediamine tetraacetic acid BD Vacutainer tubes (Franklin Lakes, New Jersey) and centrifuged at 1250 × *g* for 10 min, and plasma was removed, aliquoted, and frozen at −80°C. Plasma IL-6, IL-1 receptor antagonist, and tumor necrosis factor alpha were assessed using high-sensitivity enzyme-linked immunosorbent assays (R&D Systems, Abingdon, United Kingdom). Limits of detection were .039 pg/mL, 6.26 pg/mL, and .038 pg/mL with associated intra-assay and interassay coefficients of variation of 7.4% and 7.8%, 5.3% and 8.6%, and 5.3% and 8.4%.

### Spatial Memory Task

UnrealEngine2 Runtime software (Epic Games, Cary, North Carolina) was used to present a first-person perspective of a plane surrounded by a circular cliff (virtual diameter 180 m). Background mountains, clouds, and the sun (created using Terragen; Planetside Software, Cheshire, United Kingdom) projected at infinity were used to provide orientation cues. Two separate counterbalanced arenas with grassy or rocky planes and differently rendered mountains and clouds were used for the two encoding sessions. Participants explored the arena for 2–3 min using right-handed button presses to move forward, left, and right. Then 16 unique objects were sequentially presented within the arena, and participants were instructed to remember their identity and spatial location before picking them up. After all objects were acquired, participants performed free recall of the object identities. They then returned to the virtual reality environment. A picture of one object was presented, following which participants moved to where they thought the cued object had been presented, indicated by a button press, and recorded their confidence (range, 1–5) for this location. For the first two sessions (T1 and T2), the object was shown again in its correct position, and participants collected it by running over it. The next object was cued after a variable intertrial interval. On the third session (T3), object recall and relocation phases were completed for objects learned at T1 (set 1) and T2 (set 2). Performance was indexed by accuracy of object spatial location: mean (1/distance from true object location in virtual meters) and number of objects recalled.

### Procedural Memory Task

Participants were asked to trace between two concentric five-pointed stars viewed in a mirror as quickly and accurately as possible. Both their hand and the concentric stars were obscured from direct view. Time taken to complete two trials was used as an index of performance.

### Image Acquisition and Analysis

The PET scans (mean 155.3 ± 11.8 MBq FDG) were acquired for 35 min on a Siemens Biograph-64 PET-CT scanner (Siemens Healthcare, Erlangen, Germany) in three-dimensional dynamic acquisition mode. Participants lay supine with eyes open. Before each PET acquisition, a low-dose computed tomography scan (120 kVp, 10 mA) was acquired for attenuation correction. After correction for scatter, random effects, and effects of attenuation, images were reconstructed in 1-min windows using Siemens proprietary iterative three-dimensional reconstruction schema (21 iterations and 8 subsets). Individual 1-min scans were realigned and summed to produce a single 35-min activation scan per session, which was coregistered to subjects’ structural magnetic resonance imaging scans and then spatially smoothed with an 8-mm full width at half maximum Gaussian kernel using standard SPM8 methods (Wellcome Trust Centre for Neuroimaging, Institute of Neurology, United College London, London, United Kingdom; http://www.fil.ion.ucl.ac.uk/spm).

Normalized images were included within a second-level flexible-factorial analysis of variance (ANOVA) (repeated factor time, baseline, 4 hours, 8 hours; between-subject factor group, early, late inflammation). Main effects of time and group and group × time interaction were included in the model. Normalization to a grand mean scaled value of 50 mL/100 g/min was applied, and global effects were included as nuisance covariates in the general linear model (analysis of covariance). Correlations between changes in resting glucose metabolism and object-location accuracy together with interactions with inflammatory status (modeled using a dummy variable) were investigated in a separate regression analysis.

Anatomic localization of MTL structures was based on Insausti *et al.* (35), Monte Carlo simulation (1000 iterations) was used to determine cluster extent thresholds for whole-brain correction at *p* < .01 (36), and a cluster threshold of >19 voxels was adopted. Regression analyses followed by Goodman test for mediation were used to investigate relationships between inflammatory challenge and changes in object-location accuracy and right parahippocampal activity Montreal Neurological Institute (24, −32, −32) between encoding sessions T1 and T2. Mediation analyses were performed using the interactive calculation tool for mediation tests (http://quantpsy.org/sobel/sobel.htm).

## Results

### Inflammatory Responses to Typhoid Vaccination

**Figure 2:**
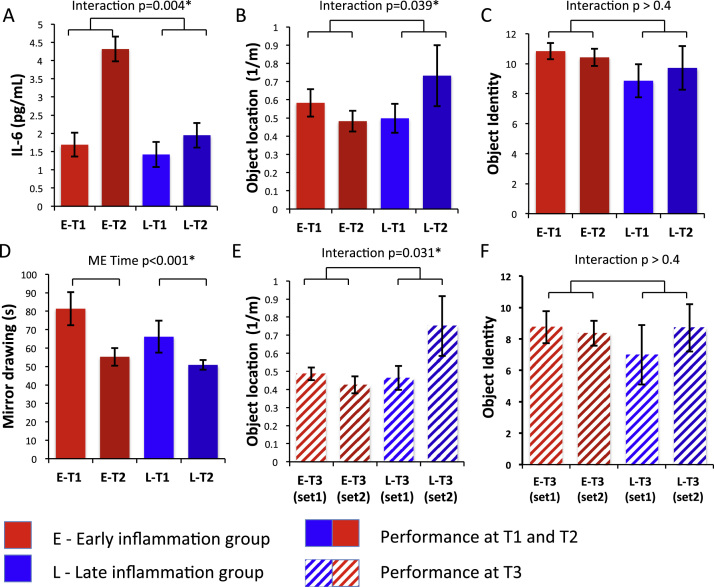
Cytokine and memory performance in the early and late inflamed groups. **(A)** Plasma interleukin-6 levels demonstrating a significantly greater increase in interleukin-6 (from session T1 to T2) in the early compared with late inflammation group. **(B)** Object location accuracy (proximity score) in units of 1/virtual meters at each encoding session (T1 and T2). Location accuracy decreased 4 hours after typhoid vaccination (early inflammation group) but increased 4 hours after placebo (late inflammation group). **(C)** Number of objects (maximum 16) correctly recalled during the two encoding sessions (T1 and T2). **(D)** Mean time taken to complete mirror tracing of a five-pointed star demonstrating a significant improvement across time in both groups. **(E)** Object location accuracy (proximity score) at the final session (T3) for objects encoded at T1 (set 1) and T2 (set 2). **(F)** Number of objects (maximum 16) correctly recalled at the final session (T3) for objects encoded at T1 (set 1) and T2 (set 2). Asterisk indicates statistical significance at *p* < .05. IL-6, interleukin-6.

Cytokine analyses confirmed significantly higher circulating inflammatory cytokines at encoding session 2 (T2) compared with encoding session 1 (T1) in the early, but not late, inflammation group (Figure 2A): group × time interaction IL-6 [*F*_1,18_ = 6.91, *p* = .017], IL-1 receptor antagonist [*F*_1,18_ = 11.77, *p* = .003]. A similar increase in IL-6 (2.82 pg/mL) was also observed after typhoid vaccination (between T2 and T3) in the late inflammation group (Figure S1 in Supplement 1). Both groups showed inflammation at T3 and absence of inflammation at baseline (T1) with only the early inflammation group inflamed at encoding session two (T2).

### Effects of Acute Inflammation on Memory Performance

Immediate recall of object identity and location at T2 and T1 demonstrated a significant group × encoding session interaction for object location but not object identity [*F*_1,17_ = 5.01, *p* = .039 and *F*_1,17_ = .66, *p* = .43]. Post hoc *t* tests also demonstrated a significantly greater reduction in proximity score across the two encoding sessions (T1 and T2) in the early inflammation group, who demonstrated inflammation at T2, compared with the late inflammation group, who did not demonstrate inflammation at this time point (−.100 vs. .234 m^−1^; *t*_17_ = 2.24, *p* = .039) (Figure 2B,C). Although performance increased from session T1 to session T2 in the late inflammation group given placebo at T1 (a practice effect), it decreased in the early inflammation group given vaccine, suggesting that inflammation impaired object-location encoding during the T2 session. This effect was maintained at the later recall session (T3) when both sets of objects were recalled and both groups demonstrated inflammation: group × encoding session interaction [*F*_1,17_ = 8.40, *p* = .01]. Post hoc *t* test again demonstrated a significantly greater reduction in proximity score at T3 for objects encoded at T2 compared with objects encoded at T1 in the early compared with late inflammation group (−.061 vs. .287 m^−1^; *t*_17_ = 2.90, *p* = .01) (Figure 2E,F). The impairing effect of inflammation on encoding location of objects seen at T2 was preserved when these objects were later recalled at T3.

Performance on the mirror-tracing task revealed no significant group × time interaction [*F*_1,18_ = 1.00, *p* = .33], although improved performance across time was observed in both groups [main effect of time *F*_2,18_ = 23.58, *p* < .001] (Figure 2D). These results suggest a selective action of inflammation on object-location memory that is not mediated via nonspecific effects on task motivation or response time.

To explore whether effects on object-location memory were mediated by actions at encoding or consolidation, we next performed a three-way ANOVA: group (early inflammation, late inflammation), encoding session (T1, T2), and recall session (T3 recall of first set, T3 recall of second set) with the prediction that actions at consolidation would be reflected by greater effects at late (T3) than early (T1 and T2) recall, owing to impaired consolidation in the early compared with the late inflammation group. This ANOVA confirmed the previously observed encoding session × group interaction [*F*_1,17_ = 4.44, *p* = .028]. However, no additional recall session × group or encoding session × recall session × group interactions were observed [*F*_1,17_ = .84, *p* = .45 and *F*_1,17_ = .80, *p* = .47] suggesting a predominant effect at encoding.

To address this situation further, we regressed immediate (T1 and T2) against late (T3) performance for both object sets with inclusion of a dummy variable encoding group membership. This regression demonstrated an anticipated strong dependence of late on early performance for both sets of objects [*F*_1,15_ = 6.08, *p* = .026 for object set 1 and *F*_1,15_ = 29.67, *p* < .0001 for object set 2] but no interaction with group [*F*_1,15_ = 1.53, *p* = .26 and *F*_1,15_ = .77, *p* = .39]. Finally, we performed a 2 (group) x× 2 (recall session) ANOVA on performance at T3 corrected for T1 and T2 performance. This ANOVA failed to show a significant recall session × group interaction [*F*_1,17_ = 2.16, *p* = .16]. Together, these analyses suggest a significant action of inflammation on early encoding and consolidation processes with little evidence to support additional effects on late consolidation processes. They also provide empiric support for a direct influence of inflammation on early encoding and consolidation mechanisms rather than nonspecific effects on motivation in which a greater decrement in performance at T3 compared with T2 would be expected in the late inflammation group (subjects demonstrated inflammation only at the later time point) compared with the early inflammation group (subjects demonstrated inflammation at both time points).

### Effects of Acute Inflammation on Resting Brain Glucose Metabolism

**Figure 3:**
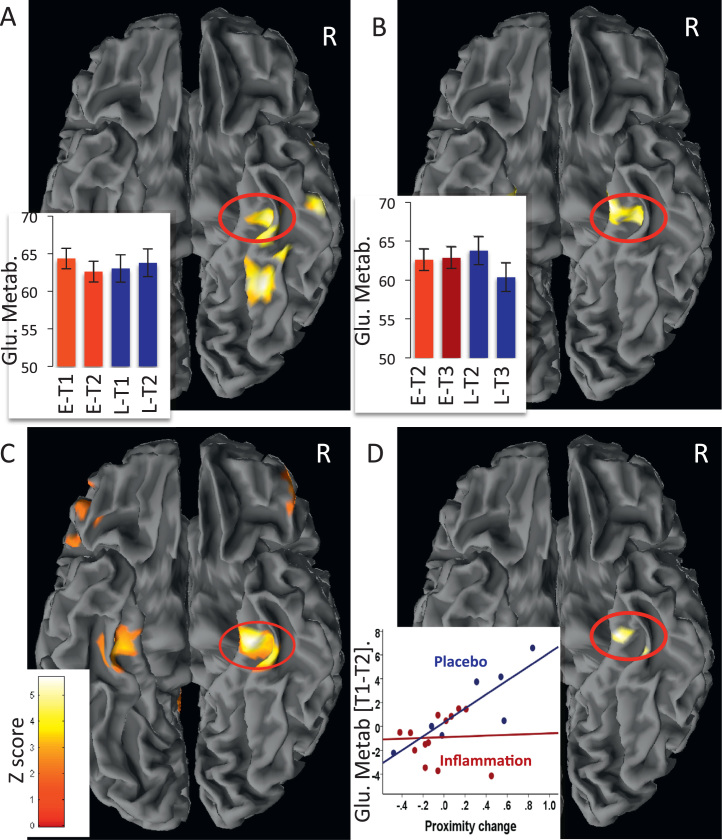
Brain regions sensitive to acute inflammation and effects on object-location encoding. **(A)** Regions showing a greater reduction in glucose metabolism after inflammation compared with placebo between sessions 1 and 2. Contrast shown in T1 - T2 early group minus T1 - T2 late group. **(B)** Regions showing a greater reduction in glucose metabolism after inflammatory challenge in the late compared with early inflamed group between sessions 2 and 3. Contrast shown in T2 and T3 early group compared with T2 and T3 late group. The y axis in **(A)** and **(B)** shows estimated glucose metabolism in mL/100 g/min. **(C)** Regions showing a positive correlation between change in object-location accuracy and change in glucose metabolism between the two encoding sessions (T1 and T2) across all participants. **(D)** Medial temporal lobe region showing a significant group × location accuracy interaction between the two encoding sessions (T1 and T2). The y axis shows change in glucose metabolism between T1 and T2 in mL/100 g/min. E, early inflammation group (received vaccine after first scan); L, late inflammation group (received vaccine after second scan 2).

Analysis of PET data (T1 and T2 in the early compared with the late inflammation group) demonstrated a reduction in glucose metabolism within a discrete cluster of regions focused on the right parahippocampal and perirhinal cortex 4 hours after inflammation compared with placebo (Table 1; Figure 3A). These regions all survived whole-brain correction at *p* < .01. This finding was replicated 4 hours after inflammatory challenge in the late compared with early inflammation group (T3 compared with T2) (Table 1; Figure 3B), robustly demonstrating acute sensitivity of MTL structures to peripheral inflammation.

**Table 1:** Brain Regions Showing an Acute Reduction in Resting Glucose Metabolism After Inflammatory Challenge

Side	Region	Coordinates	*Z* Score	Cluster	*p*_uncorrected_	*p*_corrected_
Inflammation-Induced Reductions in Glucose Metabolism (Early Inflammation Group)						
R	Parahippocampus/perirhinal	(36 −28 −24)	3.73	345	<.001	<.01
R	Fusiform gyrus	(32 −54 −14)	3.89	190	<.001	<.01
R	Inferior temporal gyrus	(57 −18 −33)	3.56	76	<.001	<.01
R	Temporal pole	(57 11 −11)	3.42	28	<.001	<.01
Inflammation-Induced Reductions in Glucose Metabolism (Late Inflammation Group)						
R	Entorhinal/perirhinal	(21 −19 −27)	3.50	117	<.001	<.01
L	Entorhinal/perirhinal	(−24 −13 −26)	3.67	71	<.001	<.01
R	Parahippocampus/perirhinal	(33 −24 −24)	3.22	10	<.001	

L, left; R, right.

**Figure 4:**
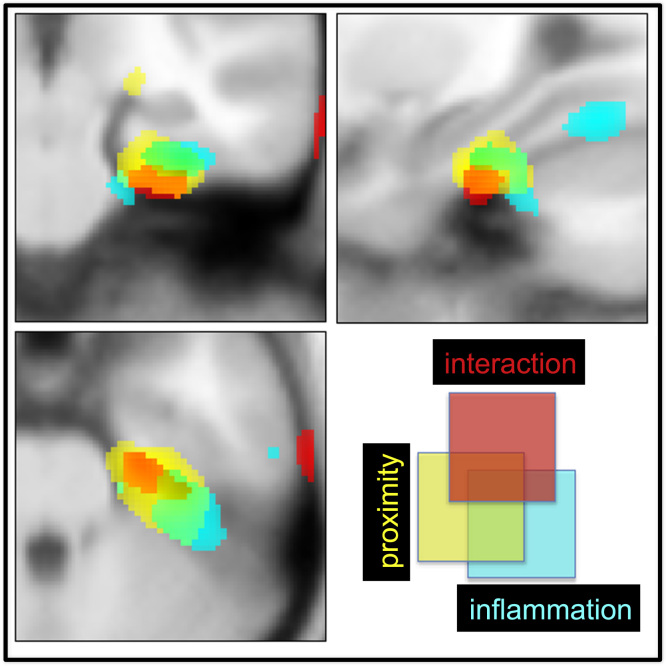
Right medial temporal lobe regions sensitive to inflammation, change in object-location accuracy, and interactions with inflammation. Cyan indicates regions showing a reduction in glucose metabolism after inflammation (T1 - T2 early group minus T1 - T2 late group). Yellow indicates regions showing a positive correlation between change in object-location accuracy (T1 and T2) and change in glucose metabolism (T1 and T2) across all participants. Red indicates area showing group × accuracy interaction—that is, the region mediating the effects of group membership (inflammation status) on change in accuracy for encoding object location.

**Figure 5:**
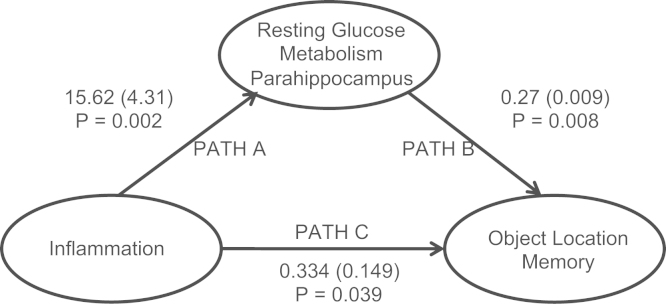
Mediation analysis showing that the changes in parahippocampal glucose metabolism mediate the effects of inflammation on memory for object location. Path coefficients (standard error of path coefficients) are shown for each path of the mediation model.

To investigate whether this change in glucose metabolism between encoding sessions predicted changes in object-location accuracy. we next performed a regression analysis on the PET data (i.e., T1 and T2 metabolism vs. T1 and T2 accuracy) (Table 2). This analysis revealed striking correlations between activity change in bilateral parahippocampal and rhinal cortex and change in object-location accuracy across all participants (Figure 3C)—that is, there was a general relationship between change in parahippocampal and rhinal glucose metabolism and change in object-location accuracy. However, repetition of this analysis after inclusion of an interaction term coding group membership (early or late) also revealed a discrete contiguous region within the right parahippocampal gyrus that mediated the detrimental effects of inflammation on object-location encoding (Figures 3D and 4). In other words, inflammation disrupted the relationship between parahippocampal metabolism and subsequent accuracy for object-location encoding. This interpretation was supported further by mediation analysis, which showed that inflammation induced changes in right parahippocampal glucose metabolism (T1 and T2) Montreal Neurological Institute (24, −21, −32) that significantly mediated effects of inflammation on object-location memory (T1 and T2 accuracy) (Goodman test = 3.58 [SE .74], *p* < .00035) (Figure 5).

**Table 2:** Brain Regions in Which Changes in Blood Glucose Metabolism Between the Two Encoding Sessions (T1 and T2) Predicted Associated Changes in Memory for Object Location

Side	Region	Coordinates	*Z* Score	Cluster	*p*_uncorrected_	*p*_corrected_^a^
Regions Showing Positive Correlations Across All Participants						
R	Parahippocampus/perirhinal	(27 −22 −27)	4.19	751	<.001	<.01
L	Parahippocampus/perirhinal	(−29 −28 −26)	3.38	80	<.001	<.01
R	Precuneus	(15 −60 45)	3.68	370	<.001	<.01
L	Inferior parietal lobule	(−20 −51 54)	3.65	829	<.001	<.01
R	Inferior parietal lobule	(33 −46 56)	3.31	99	<.001	<.01
L	Supplementary motor area	(−15 −4 63)	3.55	95	<.001	<.01
L	Paracentral lobule	(−3 −31 63)	3.36	221	<.001	<.01
R	Mid-frontal gyrus	(35 3 46)	3.21	26	<.001	<.01
R	Mid-orbitofrontal gyrus	(48 50 −9)	3.17	25	<.001	<.01
Medial Temporal Lobe Region Showing Interaction with Group						
R	Parahippocampus/perirhinal	(24 −21 −32)	2.44	342	<.05	<.01^b^

L, left; R, right.

a *p*_corrected_ = cluster survives whole-brain correction.

b Survives correction for a medial temporal lobe region of interest.

## Discussion

Systemic inflammation is associated with selective impairment in human spatial memory but not MTL-independent procedural memory (37). Deficits in spatial memory were observed for objects learned and recalled during systemic inflammation but not objects learned in the absence of inflammation and recalled under inflammatory conditions. This study suggests a predominant effect of inflammation on early encoding and consolidation processes rather than late consolidation and demonstrates a relative absence of state-dependent effects. In rodent contextual fear conditioning paradigms 5, 23, inflammation impairs spatial memory despite being induced after visuospatial information has been attended to (and encoded) suggesting that our data are likely mediated via an action on early consolidation processes. Although our analyses failed to demonstrate significant late consolidation effects, Figure 2B and E demonstrates a nonsignificant reduction in performance at T3 compared with T1 in the early (early T3 [set 1] compared with early T1) but not late inflammation group (late T3 [set 1] compared with late T1) consistent with a potential effect on late consolidation.

Resting glucose metabolism, particularly change in bilateral parahippocampus and perirhinal cortex metabolism immediately before task performance, predicted change in accuracy across encoding sessions across all participants (Figures 2C and 3). However, this relationship was critically modulated by systemic inflammation. Within 4 hours of inflammatory challenge, glucose metabolism decreased within perirhinal and entorhinal cortex and parahippocampus (Table 1). This effect was replicated in participants challenged after the first (Figure 3A) and second (Figure 3B) scanning sessions. A discrete subregion centered on the right parahippocampus also predicted and mediated inflammatory effects on subsequent object-location memory (Figures 3D and 5). Together, these data demonstrate sensitivity of human MTL structures, notably parahippocampus, to systemic inflammation and provide mechanistic insight relevant to a broader literature linking severe or chronic inflammation to the attrition of human memory.

Studies investigating effects of inflammation on rodent spatial memory to date have predominantly focused on actions on the hippocampus 1, 5, 15, 16, 17. We did not identify a major change in hippocampal glucose metabolism after inflammation or any association between hippocampal glucose metabolism and subsequent memory performance. Although null results are hard to interpret, learning object locations relative to the boundary in this task correlates with functional magnetic resonance imaging signal from both right hippocampal and parahippocampal regions (32), suggesting greater sensitivity to detect metabolic changes in the parahippocampus. In addition, there is good evidence to suggest strong parahippocampal involvement in this type of task. Neuropsychological studies show that human performance on homologues of the Morris water maze and direct tests of object-location memory can be more strongly dependent on right parahippocampal than hippocampal integrity 37, 38, 39. Studies demonstrate a central role for the right parahippocampus in human object-location memory and support our current finding of a critical role for the parahippocampus in mediating inflammation-induced spatial memory impairments.

Right parahippocampal activity during object-location encoding has also been shown to predict subsequent retrieval success with a spatial cue (40). In monkeys, one-trial memory for object-place associations (similar to our current task) appears to be critically dependent not on hippocampus but on posterior parahippocampus (41). The contribution of the parahippocampus to within-scene object location and context memory is also dissociable from the role of perirhinal cortex in object perception and memory 42, 43, 44. In rodents, perirhinal neurons respond selectively to objects and their previous occurrence 45, 46 with selective lesions impairing performance on tasks requiring whole-object information (47). In contrast, rodents with postrhinal (parahippocampus) cortex lesions show impairment on tasks sensitive to object location but not identity (48). Similar functional distinctions between perirhinal and parahippocampal activity are also apparent in humans, with parahippocampal cortex active during object-location encoding and perirhinal cortex active to objects alone (49). Our data suggest that systemic inflammation may serve as a transient parahippocampal lesion resulting in a discrete impairment in object-location memory.

The cellular mechanisms mediating this selective impairment of human MTL function are unclear, although these mechanisms may be usefully informed by rodent studies. For example, IL-1 has been shown to reduce basal synaptic activity and synaptic transmission in a manner dependent on gamma-aminobutyric acid (50). It impairs LTP both dependent on and independent of *N*-methyl-D-aspartate 1, 2 and can decrease LTP-associated glutamate release within the dentate gyrus (51). Although these effects are currently demonstrated only in rodent hippocampus, operation of either mechanism within human parahippocampus or perirhinal cortex could conceivably contribute to the observed reduction in glucose metabolism. Local or peripheral inflammation can also impair hippocampal neurogenesis in proportion to the associated increase in microglial activation (4). However, given the time course of this effect, it is unlikely to have contributed to our results. Perhaps more pertinent is the role of neurally mediated mechanisms. Peripheral inflammation has been shown to increase rapidly activity within vagus nerve projection areas in both rodents and humans 29, 30, including insular cortex, a region that in primates has direct neural connectivity to perirhinal and parahippocampal cortex (12), areas that provide the vast bulk of inputs into entorhinal cortex and the hippocampal formation. Electrical stimulation of vagus nerve afferents results in a rapid increase in IL-1β expression within the hippocampus (31). Activation of neurally mediated immune-brain communicatory pathways may potentially modulate memory processes even in the absence of significant signaling of inflammation across the blood-brain barrier at the endothelium (26) or circumventricular organs (25).

A concern from rodent studies is that apparent effects of inflammation on learning and memory may be confounded by actions on psychomotor speed (52). We also previously reported psychomotor slowing after typhoid vaccination (53). However, our current data strongly argue against a purely psychomotor explanation for our effects. In particular, vaccination did not change time taken to relocate objects; the late inflammation group showed no decrement in recall performance at time three, and mirror-tracing task performance was unimpaired by inflammation. As such, our data support and reinforce the interpretation of rodent studies.

One unresolved question is why we did not observe an effect on object identity memory, especially given reduced glucose metabolism across an expansive MTL region encompassing perirhinal cortex. Although participants did not perform at ceiling, the relatively small number of exemplars may have reduced variability associated with this measure, and consequently it may have been insensitive to subtle changes in object identity memory. This interpretation is also suggested by data from studies that show more global reductions in memory after potent inflammatory challenges with lipopolysaccharide, which have evoked decreased immediate verbal recall of story items, immediate and delayed spatial figural features, and word list learning (54). In another study, using low-dose lipopolysaccharide challenge, declarative memory impairment was also inversely correlated with IL-6 levels (55).

Our study identifies a mechanism through which peripheral inflammation affects human spatial memory. This study has important implications for understanding how chronic inflammation exacerbates age-related cognitive decline and plausibly the increased risk of neurodegenerative disorders such as Alzheimer’s disease. Increased inflammatory markers are observed in the MTL of patients with age-related cognitive decline and Alzheimer’s disease (56). The profile of memory impairment observed in Alzheimer’s disease—selective impairment of MTL-dependent memory including impaired spatial memory (57) with often striking preservation of procedural memory (58)—is similar to what we describe here. Nevertheless, it is uncertain whether they are the cause of cognitive symptoms or a consequence of a primary disease process. Increased circulating proinflammatory cytokines have been associated within an increased risk of cognitive decline in both cross-sectional and prospective epidemiologic studies (8). Similarly, acute infections requiring admission to the intensive care unit convey a significantly greater risk of subsequent cognitive decline compared with other causes of intensive care unit admission (7). In healthy middle-aged adults, levels of circulating inflammatory cytokines are linked to the volume of MTL structures, specifically hippocampus (59).

In conclusion, our data suggest that MTL structures are acutely sensitive to peripheral inflammation with consequent functional impairment. Peripheral inflammation results in an acute reduction in resting MTL glucose function associated with an acute decline in human spatial memory. This knowledge is motivation for further investigation into the cognitive consequences of chronic or severe infections and inflammation.
