# Supplementary material for: Peripheral Inflammation Acutely Impairs Human Spatial Memory via Actions on Medial Temporal Lobe Glucose Metabolism
Source: Biol Psychiatry. 2014 Oct 1;76(7):585–93. doi: 10.1016/j.biopsych.2014.01.005 (PMC4166523; doi:10.1016/j.biopsych.2014.01.005)
Supplement: Supplementary file 1 — Supplementary Material [file mmc1.pdf]

Peripheral Inflammation Acutely Impairs Human Spatial Memory via Actions on Medial Temporal Lobe Glucose Metabolism

Supplement 1

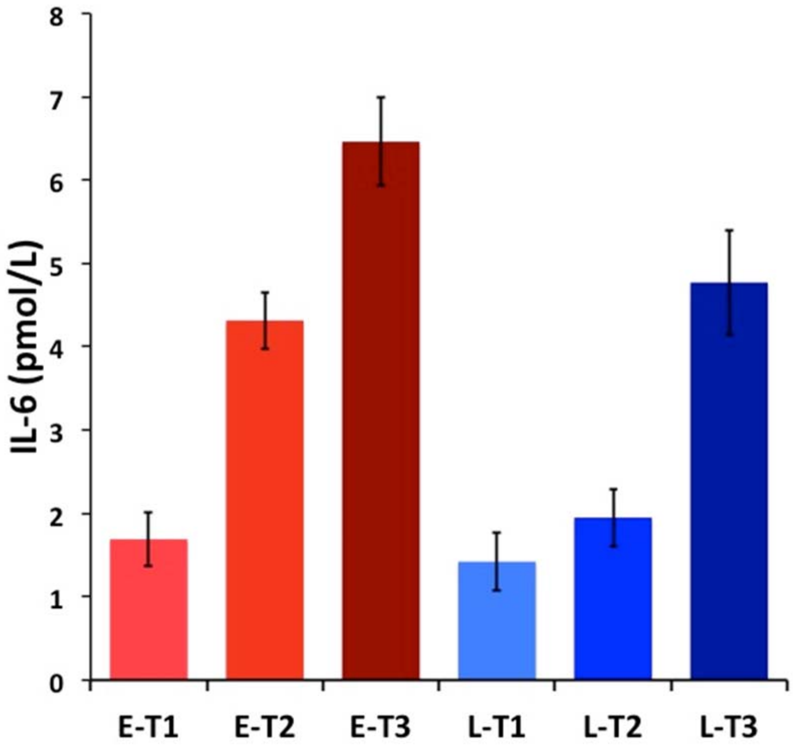

Figure S1. Interleukin (IL) -6 levels at testing sessions T1, T2 and T3 in the early (E) inflammation group who received typhoid vaccination after T1 and the late inflammation (L) group who received typhoid vaccination after T2.
